# Analysis of Memory Antibody Responses in Individuals with Zika-Associated Guillain–Barré Syndrome

**DOI:** 10.3390/v16111704

**Published:** 2024-10-30

**Authors:** Michelle Premazzi Papa, Grace Mantus, Kareem Kabra, Carlos Herrera Gomez, Adam Ward, Liliana Encinales, Andres Cadena, Aileen Chang, Rebecca M. Lynch

**Affiliations:** 1 Department of Microbiology, Immunology, and Tropical Medicine, The George Washington University, Washington, DC 20037, USAchang@email.gwu.edu (A.C.); 2 Department of Medicine, The George Washington University, Washington, DC 20037, USA; 3 Allied Research Society Colombia, Barranquilla 080020, Atlántico, Colombia; 4 Clínica de la Costa SAS, Barranquilla 080020, Atlántico, Colombia

**Keywords:** Dengue, Zika, Guillain–Barré Syndrome, neutralizing antibodies, pro-inflammatory cytokines, E dimer epitope, N154A, glycan

## Abstract

The Zika virus (ZIKV) was responsible for a major outbreak in 2015 in the Americas. Infections were associated with increased cases of microcephaly in infants and Guillain–Barré Syndrome (GBS) in adults. Our group previously demonstrated that Zika-associated GBS correlated with the increased neutralization of ZIKV and DENV2, but the antibody specificity was not analyzed. Here, we generated reporter virus particles (RVPs) of ZIKV with specific-point mutations that allowed us to investigate the specificity of circulating plasma antibodies at two different timepoints from individuals with Zika-associated GBS. We found that neutralizing antibody titers to ZIKV waned between one and two years post-ZIKV infection in GBS-negative but not GBS-positive individuals. Interestingly, plasma neutralization by GBS-negative individuals was more sensitive to a mutation at position N154A than plasma from GBS-positive individuals. To determine if waning was associated with different levels of B-cell activation at the time of infection, pro-inflammatory cytokines were measured, but no differences were observed in people with or without GBS. These data suggest subtle differences between GBS-positive and-negative individuals’ circulating antibodies, where antibodies from GBS-positive individuals may target different epitopes and remain in circulation longer as compared to GBS-negative individuals.

## 1. Introduction

In 2015, an outbreak of Zika virus (ZIKV) emerged in the South and Central Americas, causing thousands of infections [1]. ZIKV is an arthropod-borne virus that belongs to the Flaviviridae family, genus Flavivirus. Flaviviruses are characterized by an icosahedral arrangement of 90 Envelope (E) protein heterodimers on their surface [2,3], with each E protein comprised of three domains (DI, DII, and DIII). E proteins of both DENV and ZIKV share 54–59% amino acid sequence identity, and while both have a glycosylation site at positions N153/154 [4], only the DENV E protein contains another glycosylation site at position N67 [5]. These glycans are frequently found to be targeted by neutralizing antibodies [6]. Anti-ZIKV and DENV-neutralizing epitopes have been well-defined through the characterization of monoclonal antibodies (mAbs). Antibodies isolated to date target all three domains of the E protein, including the fusion loop (FL), which is targeted by mAb 2A10G6 [7], the DIII lateral ridge targeted by ZV-67 [8], and E dimer epitopes (EDE), which are targeted by mAbs C8, A11, and C10 [9,10]. This last category of antibody (EDE) is affected by the presence or absence of the N153/N154 glycan. Because of the genetic similarity and endemic prevalence of both viruses, cross-reactive antibodies are frequently elicited, and mAbs 2A10G6, C8, A11, and C10 are all cross-reactive to both DENV and ZIKV.

Since the Zika outbreak, multiple Dengue outbreaks have occurred. Despite their genetic similarity, the Zika outbreak was associated with neurological manifestations, including microcephaly in babies and Guillain–Barré Syndrome (GBS) in adults, not commonly observed with endemic Dengue [11,12]. GBS is acute paralysis that affects thousands of people worldwide every year [13], and it is usually reported after respiratory infections caused by viruses or bacteria or after vaccination [14,15]. Analysis of the 2013 Zika outbreak in French Polynesia found a high level of association between GBS diagnosis and ZIKV infection (93% seropositivity) [16]. An increase in the number of GBS cases was also observed during the 2015 Zika outbreak in Colombia, specifically in Barranquilla [17]. Studies in Colombia showed an association with 97% of individuals diagnosed with GBS having developed Zika symptoms within the previous week (the median was 7 days) [18]. Prior work has suggested that GBS is an antibody-mediated process triggered by molecular mimicry of the microorganisms to the structural components of peripheral nerves [13], and there are studies suggesting that this same phenomenon may be occurring during ZIKV infection [19].

Our group has previously observed that people who develop GBS after ZIKV infection had higher neutralizing antibody titers against ZIKV and DENV2 within the first-month post-infection [20], although any mechanistic link remains unknown. A clinical follow-up revealed that, although GBS symptoms in most individuals are resolved within a year, for a minority, the symptoms remained for at least two years post-infection [21]. Anti-ZIKV antibodies wane over time in non-GBS individuals [22]; however, the link between higher anti-viral antibody titers and GBS remains unclear. Here, we investigate if the high neutralization titers observed in individuals with Zika-associated GBS persisted over time. We further investigated if the antibodies from individuals with Zika-associated GBS targeted different epitopes as compared to Zika-infected GBS-negative individuals.

## 2. Materials and Methods

### 2.1. Participants

Twenty-one individuals were selected from a previously described long-term study of people who did or did not develop GBS after the 2015–2016 ZIKV outbreak in Barranquilla, Colombia [21]. These individuals volunteered plasma samples in 2017 and 2018. All participants were serologically diagnosed with a ZIKV infection. Positivity was defined as an NT90 > 50 to ZIKV-H/PF, as measured in an RVP neutralization assay. The NT90 is the reciprocal plasma dilution resulting in a 90% reduction in infectivity. Twelve of these twenty-one were clinically diagnosed with GBS and met the Brighton criteria level 1 or 2 of GBS diagnostic certainty [21]. Here, we further classified as severe GBS individuals who required mechanical ventilation and received treatment in the ICU (n = 8; 66.67%). The median days of treatment in the ICU were 15.

### 2.2. Reporter Virus Particle Neutralization Assay

The neutralization of DENV1-WP (West Pac/1974), DENV2-16681 (Thailand/1984), and ZIKV-H/PF (French Polynesia/2013) by antibodies present in plasma samples was measured using a reporter virus particle (RVP) assay, as described previously [23]. Plasmids expressing DENV1-WP-CprME, DENV2-16681-CprME, ZIKV-H/PF-CprME, and the replicon plasmid WNVII-rep-REN-IB were provided by Dr. Theodore Pierson. In brief, the plasma was 5-fold-serially diluted, starting at 1:50. Samples were incubated with RVP for 1 h at 37 °C and then added to target Vero cells. Input virus dilution was calculated from titration experiments to ensure a sufficient luciferase output within the linear portion of the titration curve. Cell-only and virus-only controls were included in each plate. After 48 h, luciferase activity was measured, and neutralization curves were calculated by averaging luciferase units from triplicates, subtracting a cell-only control background, and calculating the percentage difference in the samples to virus-only controls. Data were fit by a nonlinear regression using the asymmetric 5-parameter logistic function in GraphPad Prism. The 50%, 80%, and 90% neutralizing titers (NT50, NT80, and NT90, respectively) were defined as the reciprocal plasma dilution resulting in a 50%, 80%, or 90% reduction in infectivity.

### 2.3. E Protein Specific Point Mutations

The following point mutations were made in the E protein of the ZIKV-H/PF-CprME plasmid using QuikChange Lightning Site-Directed Mutagenesis Kit (Agilent, Santa Clara, CA, USA) and specific primers as follows: K394A forward 5′-GTGGTGGGTGATCTTCGCCTCCCCGACTCCTATG-3′, reverse 5′-CATAGGAGTCGGGGAGGCGAAGATCACCCACCAC-3′; N154A forward 5′-CATGTCCTGTGTCAGCAACGATCATCCCACTGTGCTGGG-3′, reverse 5′-GTGGGATGATCGTTGCTGACACAGGACATGAAACTGATG-3′; D67N A69T forward 5′-CATCAATATCGAACATGACTTCGGACAGCCGCTGCCCAAC-3′, reverse 5′-GCGGCTGTCCGAAGTCATGTTCGATATTGATGCCTC-3′; Q77A forward 5′-CGGACAGCCGCTGCCCAACAGCAGGTGAAGCCTAC-3′, reverse 5′-GTCAAGGTAGGCTTCACCTGCTGTTGGGCAGCGGC-3′.

### 2.4. Cytokines

Cytokines were measured in plasma samples using Bio-Plex Pro Human Th17 Cytokine Panel 15-plex (Bio-Rad, Hercules, CA, USA) according to the manufacturer’s instructions. A total of 15 cytokines were analyzed: IL-1β, IL-4, IL-6, IL-10, IL-17A, IL-17F, IL-21, IL-22, IL-23, IL-25, IL-31, IL-33, IFN-γ, sCD40L, and TNF-α. Briefly, the samples were diluted 1:2 and 1:4 and incubated with capture antibody-coupled beads for 1 h. Next, the samples were washed and incubated with biotinylated detection antibodies for 30 min. This was followed by a wash and incubation with a Streptavidin-phycoerythrin conjugate (SA-PE) for 10 min. After a final wash, fluorescence was detected using the Bio-Plex 200 System (Bio-Rad), and data were analyzed using the Bio-Plex Manager 6.1.1 software.

### 2.5. Statistical Analysis

Statistical tests were performed using GraphPad Prism v. 10.0.3. *p*-values < 0.05 were considered statistically significant. The threshold for significance was 0.05.

## 3. Results

### 3.1. Demographic Characteristics of Participants

A previous study of the long-term clinical outcomes of Zika-associated GBS collected plasma from participants one and two years post-ZIKV infection [21]. All twelve individuals with GBS were diagnosed based on the Brighton criteria. Controls, who were ZIKV-infected but not diagnosed with GBS, were matched for age and geographic location but not gender, as all nine controls were female. For all participants in this study, the median time post-ZIKV infection for the first timepoint (Year 1) was 1.20 years. The median time post-ZIKV infection for the second timepoint (Year 2) collected was 2.15 years (Table 1).

### 3.2. ZIKV Titers Remain Stable over Time in GBS-Positive Individuals

**Figure 1:**
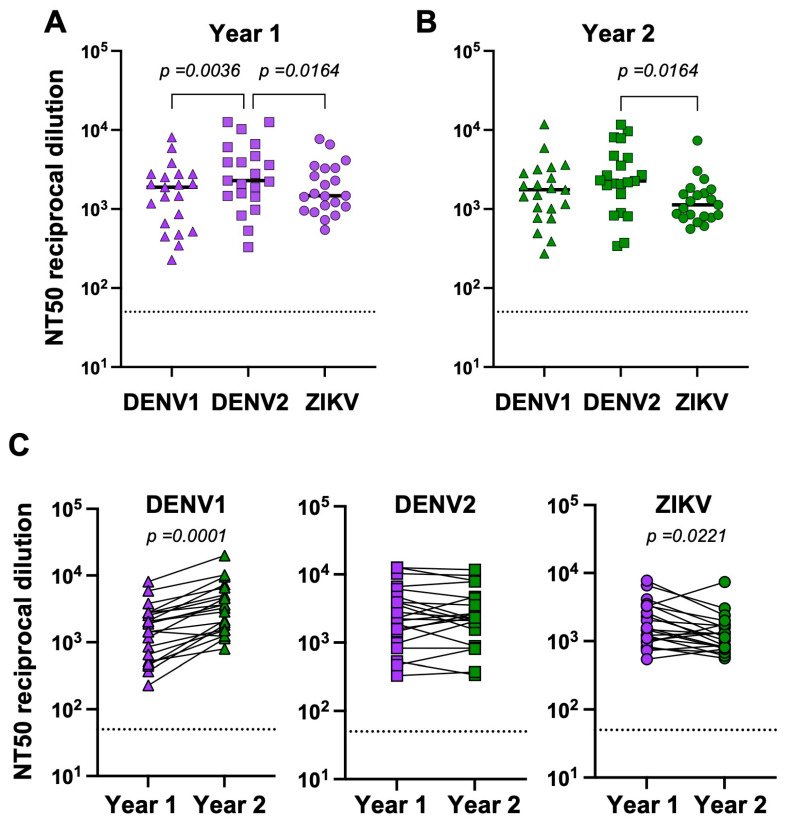
NT50 titers to DENV1, DENV2 and ZIKV. For all 21 participants, NT50 titers to the three viruses were measured at Year 1 (**A**) and Year 2 (**B**) post-ZIKV infection. ANOVA nonparametric Friedman multiple comparisons test was used. NT50 comparison between Year 1 and Year 2 timepoints for each virus was made using Wilcoxon matched pairs signed rank test (**C**). Year 1 titers are colored purple while Year 2 are colored green. DENV1 titers are indicated as triangles, DENV2 as squares and ZIKV as circles. Dotted lines indicate limit of detection of the assay. *p*-value is indicated. Black lines indicate medians.

We first characterized the durability of neutralization responses to DENV and ZIKV in this cohort. Neutralizing titers to DENV1-WP, DENV2-16681, and ZIKV-H/PF were measured at Year 1 and Year 2 timepoints. The NT50 against all three viruses tested was detectable in all participants at both timepoints. In Year 1, the medians for DENV1, DENV2, and ZIKV were 1893, 2296, and 1470, respectively. In Year 2, the medians were 1756 for DENV1, 2259 for DENV2, and 1132 for ZIKV. In Year 1, ZIKV and DENV1 titers were significantly lower than DENV2 titers (*p* = 0.0164 and *p* = 0.0036, respectively) (Figure 1A). In Year 2, ZIKV titers remained significantly lower than DENV2 (*p* = 0.0164), but DENV1 titers did not (Figure 1B). Next, we measured the differences in NT50 titers for each virus over time to investigate how viral titers changed (Figure 1C). DENV1-neutralizing titers increased between Years 1 and 2 (*p* = 0.0001), while DENV2 titers remained stable. ZIKV NT50 waned two years after ZIKV infection (*p* = 0.0221). These data reflect the overall waning of ZIKV-specific titers, as binding titers also were significantly lower in Year 2 compared to Year 1 (*p* = 0.0001) (Supplementary Figure S1A). These data are consistent with the fact that, between 2015 and 2019, there was a shift from DENV2 to DENV1, which was the predominantly circulating serotype in Colombia [24].

**Figure 2:**
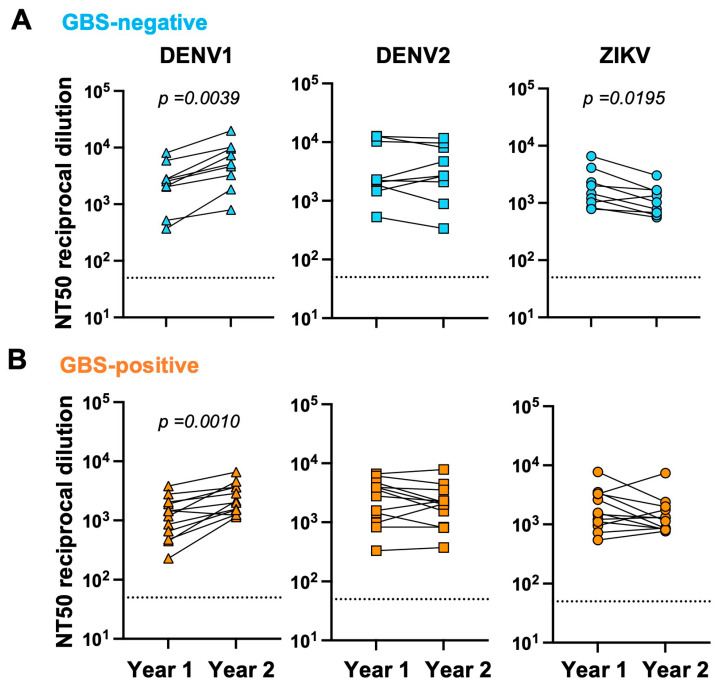
NT50 titers to DENV1, DENV2 and ZIKV. For all 21 participants, NT50 titers to the three viruses were measured at Year 1 and Year 2 post-ZIKV infection. Participants were separated into GBS-negative, light blue (**A**) and GBS-positive, orange (**B**) groups. DENV1 titers are triangles, DENV2 are squares and ZIKV are circles. Wilcoxon matched pairs-signed rank test was used for comparison. Dotted lines indicate limit of detection of the assay. *p*-value is indicated. Black lines indicate medians.

We next analyzed if there were differences in antibody titers between individuals who were GBS-negative versus GBS-positive at Year 1 and Year 2 timepoints (Figure 2A,B). Both groups significantly increased their antibody titers against DENV1 (*p* = 0.0039 for GBS-negative and *p* = 0.0010 for GBS-positive), while titers against DENV2 remained the same. Interestingly, the GBS-negative group had significantly lower ZIKV titers after two years post-infection (*p* = 0.0195), as expected with the natural waning of the immune response, while the GBS-positive group did not (*p* = 0.3013). ZIKV-specific binding titers were also significantly lower in Year 2 compared to Year 1 in both groups (*p* = 0.0039 for GBS-negative and *p* = 0.0005 for GBS-positive) (Supplementary Figure S1B).

### 3.3. Effect of GBS Clinical Severity on Antibody Titers

The differences in the ZIKV titer decline are subtle between the GBS-positive and -negative groups and could be explained by a few individuals in the GBS-positive group maintaining ZIKV titers over the years instead of slowly decreasing. We, therefore, investigated whether the clinical severity of GBS could explain differences in ZIKV titers. We defined severe GBS as spending any time in the ICU and/or having been treated with mechanical ventilation; Table 2. With this definition, eight out of twelve individuals were considered to have severe GBS, but severity (days in the ICU) did not correlate with higher antibody titers two years after ZIKV infection (Supplemental Figure S2).

### 3.4. Cytokine Levels in GBS-Positive and -Negative Groups

**Figure 3:**
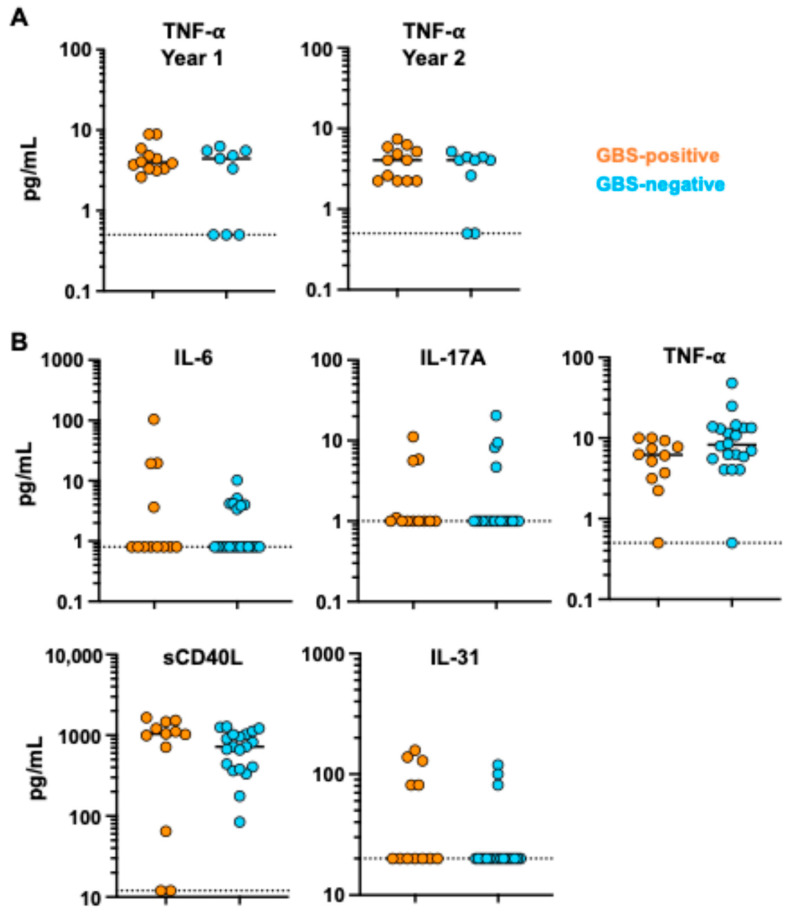
GBS-positive and GBS-negative participants have similar levels of cytokines. Fifteen-plex cytokines were measured in serum samples from 21 participants in both Year 1 and Year 2 timepoints (**A**) and from 12 GBS-positive participants (orange) and 20 GBS-negative participants (blue) with recent ZIKV infection (**B**). The nonparametric Mann-Whitney test was used to compare groups. Dotted lines indicate the limit of detection of each cytokine. Black lines indicate medians.

To investigate if maintaining antibody levels over time was associated with different levels of B-cell activation, we analyzed circulating plasma levels of ten cytokines relevant for B-cell proliferation, activation, and antibody production: IL-4, IL-6, IL-10, IL-17A, IL-17F, IL-21, IL-33, IFN-γ, sCD40L, and TNF-α [25,26,27,28,29,30,31]. TNF-α was the only cytokine detected in both timepoints, but no differences were observed between the GBS-positive and GBS-negative groups after one or two years of infection (Figure 3A). Given these timepoints were collected 1–2 years after acute infection, we wanted to analyze samples taken closer to the infection time to determine if there were differences in the B-cell activation or inflammatory environment earlier in the infection. We obtained plasma samples collected a median of 18 days post-ZIKV infection from another cohort of individuals, some of whom were diagnosed with GBS, in the same region of Colombia as our previous cohort. We used the same cytokine panel to analyze an additional 32 individuals: 12 were diagnosed with Zika and GBS (seven females and five males with median age of 38.5 years), and 20 were diagnosed with Zika but not GBS (fourteen females and six males with median age of 44.5 years). There were detectable levels of five cytokines (IL-6, IL-17A, TNF-α, sCD40L, and IL-31) in the plasma (Figure 3B). Pro-inflammatory cytokines IL-6, IL-17A, and TNF-α, as well as sCD40L, can promote B-cell activation or proliferation and class-switching [25,26,30,31], while IL-31 is related to neuronal growth [28]. We hypothesized that these protein levels could be elevated in individuals diagnosed with Zika-associated GBS. IL-6 and IL-17A were undetectable in almost all individuals tested, except for a few individuals in both groups. TNF-α and sCD40L were detected in the majority of individuals, but no significant differences were observed between the GBS-positive and -negative groups, although sCD40L had a trend toward higher levels in the GBS-positive group. Five individuals diagnosed with GBS had detectable levels of IL-31 compared with three individuals from the GBS-negative group. Together, these data show that similar levels of inflammatory activation are occurring in both GBS-positive and GBS-negative Zika-infected individuals.

### 3.5. Specificity of Antibody Responses

We next investigated if the differences between GBS-negative and GBS-positive groups were not only in neutralization titers but also in the epitopes targeted by the antibodies. To address this question, we created point mutations in the envelope of ZIKV H/PF in four well-characterized neutralizing antibody epitopes (Table 3). We sequence-verified all plasmids and tested for antigenicity to demonstrate that the mutations only affected mAbs targeting the specified epitope (Supplementary Figure S3).

**Figure 4:**
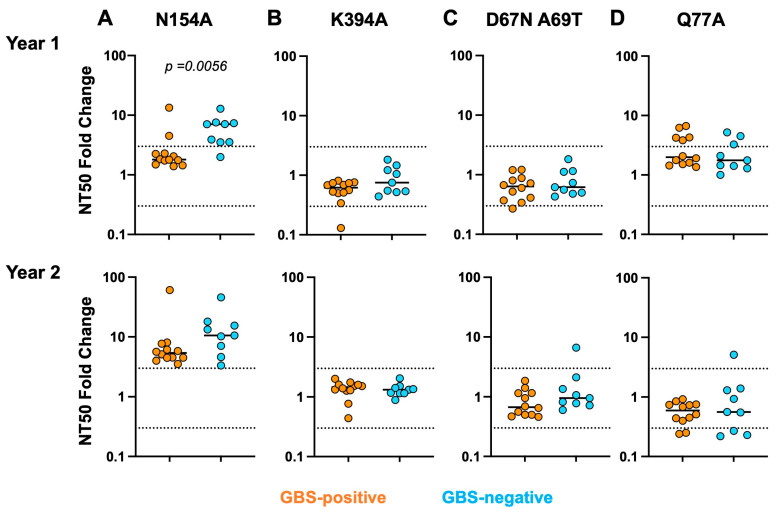
GBS-negative participants are more sensitive to a glycan removal than GBS-positive participants. NT50 fold change in N154A (**A**), K394A (**B**), D67N A69T (**C**) and Q77A (**D**) were calculated by plotting the ratio between NT50 of mutated RVP and NT50 of wild-type ZIKV H/PF RVP in both Year 1 and Year 2 timepoints. Dotted lines indicate 0.3- and 3-fold difference which marks the normal range of variation in the assay. The nonparametric Mann-Whitney test was used to compare GBS-positive group (orange) to GBS-negative group (blue). *p*-value is indicated. Black lines indicate medians.

The first mutation tested was the removal of the single glycan at position 154 within the E dimer epitope (EDE). The removal of a glycan at position N154 should result in increased neutralization sensitivity as epitopes masked by the glycan will now be available. The RVP assay results in a three-fold variation between independent experiments, and, therefore, differences in NT50 titers greater than three-fold were considered true differences in neutralization. In Year 1, almost all GBS-negative individuals neutralized ZIKV N154A better than the wild-type (WT) (greater than 3-fold, medians for the GBS-positive and GBS-negative groups are 1.80 and 7.04, respectively), but interestingly, plasma neutralization by the GBS-positive individuals was mostly unaffected by this mutation, suggesting that EDE-specific antibodies may not be dominant contributors to the response (Figure 4A). In Year 2, both groups had higher levels of EDE-directed neutralization compared to Year 1 (the GBS-positive group median is 5.37, and the GBS-negative group is 10.57), and no difference was observed between groups at this later timepoint. This finding would indicate that EDE-specific antibodies developed in the GBS-positive group between one and two years post-infection. We next investigated responses to the neutralizing epitope of the DIII lateral ridge, where a K394A mutation has been demonstrated to affect neutralization by mAbs targeting this region [8]. We observed, as have others [34], that the DIII region is not a dominant target of the antibody response as no individuals were affected by the mutation (Figure 4B).

An important difference between DENV and ZIKV E proteins is the presence of a glycan at position N67 in DENV but not ZIKV [5]. It has been shown that ZIKV-specific antibodies in a DENV-naïve rhesus macaque, frequently targeted epitopes that are exposed at this position during ZIKV infection because of the lack of glycan, and that neutralization is inhibited when a glycan at position 67 is introduced [32]. We assessed the effects of glycan 67 on the neutralization of ZIKV, and no differences were observed between the groups at any timepoint (Figure 4C). If an individual had a dominant ZIKV-specific antibody response, we would expect a decrease in the neutralization of the mutant, meaning that the fold-change in the NT50 titer between the WT and mutant would be less than three-fold. These data suggest that all individuals, regardless of GBS status, do not have detectable levels of ZIKV-specific antibodies targeting this epitope.

We analyzed the effects of the mutation Q77A, which is in the bc loop adjacent to the fusion loop region of the E protein and is an important point of contact for neutralizing antibodies [33]. Antibodies targeting the fusion loop neutralize and confer protection in ZIKV and DENV infections [7,33]. No differences in the neutralization of the ZIKV Q77A mutant were observed between both groups (Figure 4D). Taken together, these data demonstrate that there may be differences in the generation of EDE antibodies between GBS-positive and GBS-negative individuals within a year of acute infection, suggesting that subtle differences in the antibody repertoire may exist compared to GBS-negative individuals.

## 4. Discussion

Here, we analyzed the long-term plasma antibody responses in Zika-infected individuals who did or did not develop GBS. Over the two-year period, neutralizing titers to DENV1 increased within both GBS-positive and -negative individuals, likely because of dengue infections within the cohort during a potential DENV1 outbreak between Years 1 and 2 [24], while titers to DENV2 remained stable. We found that ZIKV-neutralizing titers waned from Year 1 to Year 2 after infection in the GBS-negative participants but not in people who had a concurrent GBS diagnosis. These data suggest that there is a difference in the humoral response to ZIKV in people who developed GBS. We hypothesized that the more durable ZIKV-neutralizing titers might be associated with increased inflammation or B-cell activation during the infection. We have previously shown that ZIKV-infected individuals who develop GBS have higher neutralizing titers against ZIKV and DENV2 one month post-infection [20]. Here, we analyzed the circulating cytokine profile in both GBS-positive and -negative individuals at the one- and two-year timepoints. We were only able to detect TNF-α at these timepoints and observed no difference between the groups. Given that we were analyzing timepoints one and two years after the acute infection, we also obtained plasma from GBS-positive and -negative individuals collected within 18 days of ZIKV infection. Here, we were able to detect pro-inflammatory cytokines associated with B-cell responses, but we did not observe a statistical difference between the groups. We did, however, observe a trend toward higher levels of sCD40L in the GBS-positive group. While we did not observe a difference in IL-6, Weller and colleagues have demonstrated that IL-6 is upregulated in individuals who develop GBS within 10 days of onset of symptoms [20,35]. This difference might indicate that our analysis at 18 days was too late to observe differences in the upregulation of certain cytokines, and future studies of earlier timepoints will be needed. It is also possible that the inflammatory environment was within a different tissue compartment and not detectable in the blood.

To understand if epitopes were differentially targeted between the GBS-positive and -negative groups, we investigated the specificity of the plasma responses. The characterization of virus-specific and cross-reactive antibodies has revealed conserved epitope targets on the flavivirus E protein [36]. Glycosylation sites are important for increasing virus pathogenesis [37], and glycan substitutions can affect virus infection and increase antibody neutralization [38]. The mutation N154A removes an N-linked glycosylation site, and antibodies targeting the EDE region will neutralize better once these epitopes are exposed by glycan removal. We found that one year after ZIKV infection, GBS-positive individuals had lower levels of EDE-targeting antibodies compared to GBS-negative. However, two years after ZIKV infection, both groups had similarly high levels of EDE-targeting antibodies. EDE-targeting antibodies are commonly found during DENV infection [9,39], and therefore, this observation reflects that Dengue infection occurred within the cohort during a possible DENV1 outbreak between Years 1 and 2. Mutations at positions K394A and Q77A did not significantly affect neutralization by most individuals studied here, although one person within the GBS-positive group did have reduced neutralization of the ZIKV K394A mutant virus, suggesting a dominant neutralizing response against DIII of the E protein. Thus, these epitopes do not appear to be the dominant targets of the majority of individuals’ polyclonal responses, although neutralizing mAbs that target these sites have been isolated before [40,41].

One study demonstrated that a rhesus macaque who was flavivirus-naïve before being infected with ZIKV developed ZIKV-specific neutralizing antibodies that were affected by the addition of a glycan at position 67 (D67N A69T) [32]. When we tested whether this mutation affected plasma neutralization within our cohort, we did not observe any reduced neutralization of ZIKV after the addition of the glycan, suggesting that ZIKV-specific antibodies targeting this site are not dominant within our individuals. This finding is not surprising given both the endemicity of Dengue within the region and the high neutralization titers of DENV 1 and 2 observed within the cohort, which suggest that individuals likely had a more cross-reactive antibody response (dominant toward EDE and not ZIKV-specific).

Our study provides a longitudinal analysis of a unique cohort containing twelve individuals who develop GBS after ZIKV infection. Overall, we detected subtle differences between GBS-positive and -negative individuals, with ZIKV-neutralizing titers remaining higher in the GBS-positive group two years post-infection and a lower titer of circulating EDE-targeted antibody response in the GBS-positive people at one-year post-infection. Some limitations of the study are the small sample size and the fact that GBS treatment includes plasmapheresis and IVIG, which could possibly affect the antibody repertoires. However, the samples studied here were collected one and two years post-infection and treatment, making this unlikely. Finally, a cohort of people with GBS is rare, and this study brings new insights into antibody responses in individuals with Zika-associated GBS.

## Figures and Tables

**Table 1:** Demographic characteristics of 21 Colombian individuals diagnosed with Zika.

Clinical Group	N	Female Gender n (%)	Age Range	Median Time Post-ZIKV Infection (Years)	
				Year 1	Year 2
GBS-positive	12	5 (41.67)	21–82	1.23	2.18
GBS-negative	9	9 (100)	21–73	1.18	2.12
Total	21	14 (66.67)	-	1.20	2.15

**Table 2:** Severe vs. non severe GBS.

GBS Status	PID	ICU (Days)	Mechanical Ventilation
Severe (n = 8)	1	38	Y
	15	34	Y
	16	8	Y
	24	1	N
	29	22	Y
	30	5	N
	32	7	Y
	34	27	Y
Non-severe (n = 4)	13	0	N
	25	0	N
	31	0	N
	47	0	N

Abbreviations: PID, patient ID; Y, yes; N, No.

**Table 3:** Mutations to probe for epitope specificity.

Mutants ^1^	Epitopes	Sensitive Ab	Ref
N154A	E dimer epitope	C8, C10, A11	[9]
K394A	lateral ridge	ZV67	[8]
D67N A69T	dimer-dimer	A11	[32]
Q77A	bc loop	2A10G6	[33]

^1^ Mutations on the E protein of ZIKV H/PF RVP.

## Data Availability

The raw data supporting the conclusions of this article will be made available by the authors on request.

## Supplementary Materials

The following supporting information can be downloaded at https://www.mdpi.com/article/10.3390/v16111704/s1, Figure S1: Binding IgG to ZIKV E monomers wane two years post-infection. Binding titers (EC50) to ZIKV E monomer protein was evaluated one year and two years after ZIKV infection. EC50 comparison between Year 1 and Year 2 timepoints for all participants (A) or in the GBS-negative and GBS-positive groups (B) was made using Wilcoxon matched pairs signed rank test. *p*-value is indicated. Dotted lines indicate limit of detection of the assay; Figure S2: Correlation between days in ICU and ZIKV NT50 titers. ZIKV NT50 titers from twelve GBS-positive participants were correlated with ICU days. Nonparametric Spearman correlation was used for comparison (r = 0.03, *p* = 0.9238); Figure S3: Point mutations were made on ZIKV E protein and mutated RVPs were tested using specific antibodies. Mutation at positions N154A (A), K394A (B), D67N A69T (C) and Q77A (D) were tested using monoclonal antibodies targeting EDE1 (C8 and C10); EDE2 (A11); lateral ridge (Z004 and ZV67); and fusion loop (2A10G6). Eight-point, five-fold dilution series was made, and percent neutralization was calculated. Wild-type (WT) RVP is represented by dark circles and Mutant RVP is open circles.
