# Supplementary figures and images for: Analysis of Memory Antibody Responses in Individuals with Zika-Associated Guillain–Barré Syndrome
Source: Viruses. 2024 Oct 30;16(11):1704. doi: 10.3390/v16111704 (PMC11598927; doi:10.3390/v16111704)

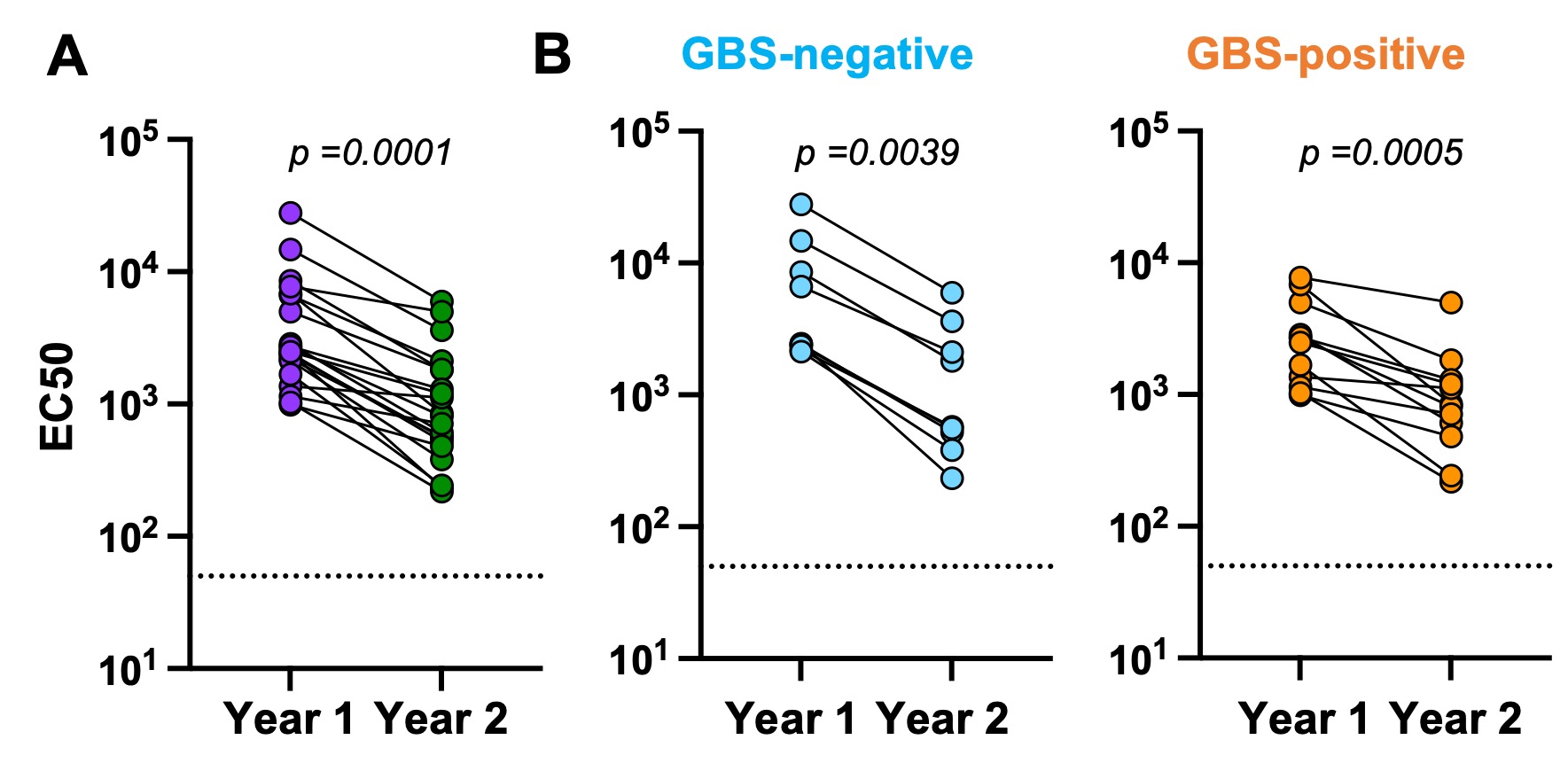

Supplement: Supplementary file 1 [file viruses-16-01704-s001.zip › Figure S1.jpg]

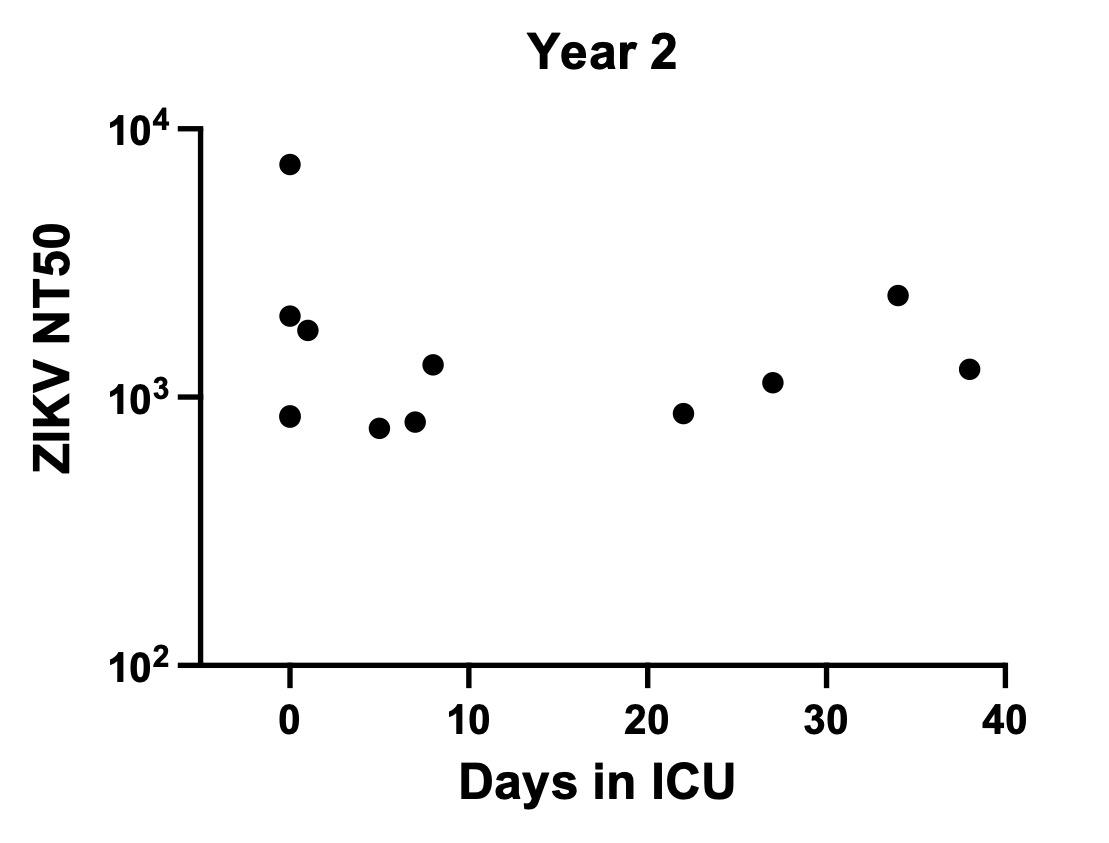

Supplement: Supplementary file 1 [file viruses-16-01704-s001.zip › Figure S2.jpg]

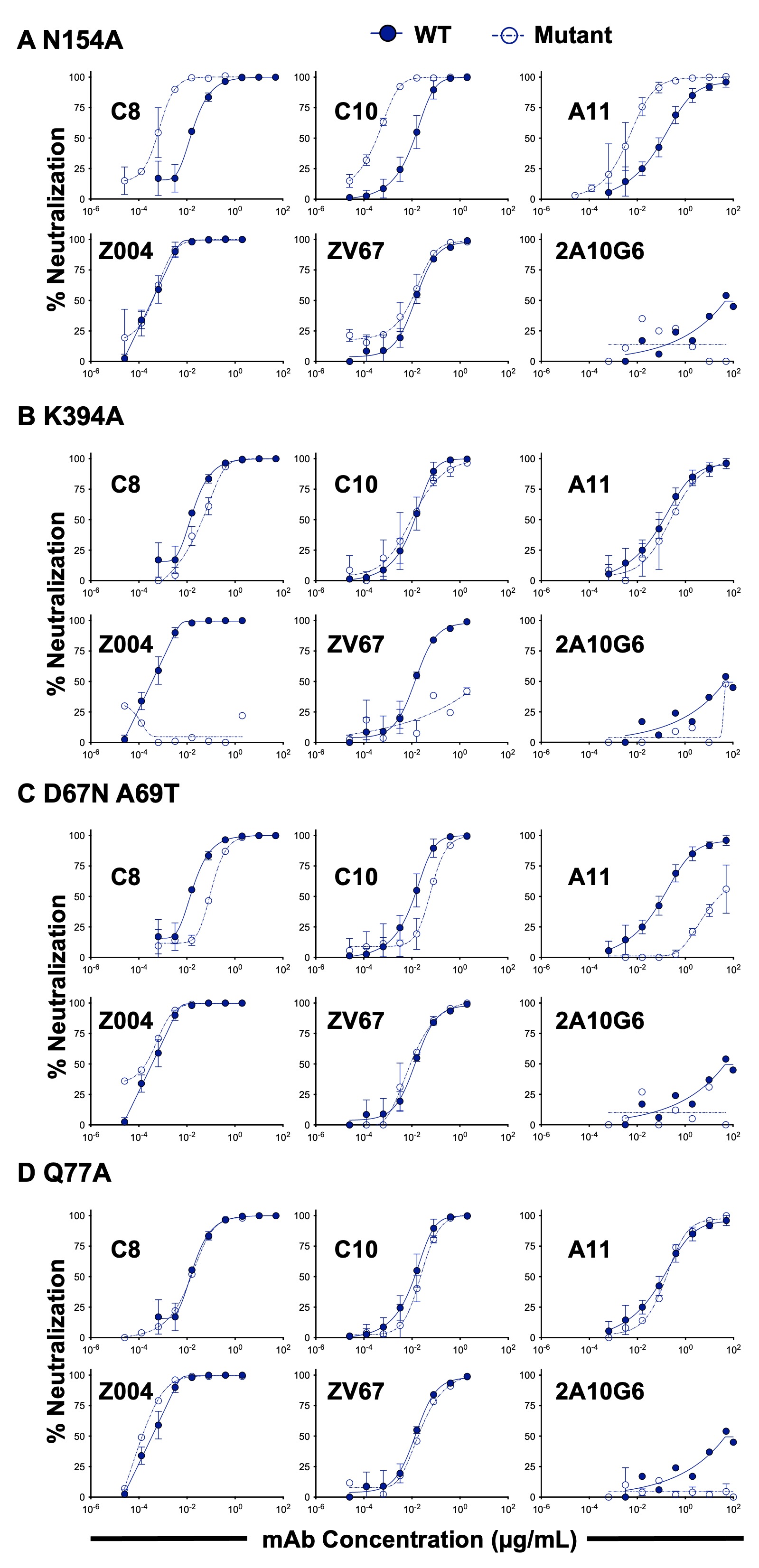

Supplement: Supplementary file 1 [file viruses-16-01704-s001.zip › Figure S3.jpg]
